# Comparison of Individual Retinal Layer Thicknesses between Highly Myopic Eyes and Normal Control Eyes Using Retinal Layer Segmentation Analysis

**DOI:** 10.1038/s41598-019-50306-w

**Published:** 2019-09-30

**Authors:** Jin Hyung Kim, Sung Hoon Lee, Jae Yong Han, Hyun Goo Kang, Suk Ho Byeon, Sung Soo Kim, Hyoung Jun Koh, Sung Chul Lee, Min Kim

**Affiliations:** 1 Institute of Vision Research, Department of Ophthalmology, Severance Hospital, Yonsei University College of Medicine, 134 Shinchon-dong, Seodaemun-gu, Seoul Korea; 2 Eyereum Eye Clinic, Seoul, Korea; 3 Department of Ophthalmology, Gangnam Severance Hospital, Yonsei University College of Medicine, 211, Eonjuro, Gangnam-gu, Seoul Korea

**Keywords:** Macular degeneration, Retinal diseases

## Abstract

The incidence of myopia is increasing worldwide, and the investigation on pathophysiology of myopia is becoming more important. This retrospective study aimed to compare the thicknesses of individual retinal layers between high-myopic and control eyes, and to evaluate the effects of age and sex on each retinal layer thickness. We assessed 164 subjects and divided them into two groups based on axial length (AL) (i.e., high-myopic group (AL ≥ 26 mm) and control group (AL < 26 mm)). Individual retinal layer thicknesses of five subfields in the macula were measured using automated retinal segmentation software packaged with the spectral-domain optical coherence tomography and were compared. In high-myopia group, the thicknesses of total retina and all individual retinal layers in central and entire perifoveal subfields were significantly thicker than the corresponding layers in control group after adjustment for ocular magnification (all P < 0.05). There were no significant effects of sex on individual retinal thicknesses, and age had less negative effects on the thicknesses of retina layers in high-myopic eyes than normal eyes. Axially elongated, non-pathologic highly myopic eyes had different structural features than control eyes, with significantly greater individual macular layer thicknesses independent of sex or age.

## Introduction

The prevalence of myopia has been increasing worldwide and the incidence is relatively greater in East- and Southeast-Asian countries (i.e., up to 80%)^1–3^. High myopia is defined as a spherical equivalent (SE) <−6 diopters or an axial length (AL) ≥26 mm^4–6^. It is associated with axial elongation of the globe and is one of the principal causes of visual impairment. Macular abnormalities and degenerative or atrophic change of the posterior segment, including macular holes, myopic macular schisis, choroidal neovascularization, chorioretinal atrophy, lacquer cracks, retinal detachment, posterior staphyloma, and epiretinal membrane are conditions associated with high myopia^7–11^. Macular anatomic abnormalities were found in up to 22% of eyes with high myopia^12^.

Myopic enlargement of the globe is mainly related to axial length prolongation and leads to retinal tissue stretching, thinning, and reduced retinal function^13–16^. These changes could be associated more significantly with individuals with a higher degree of myopia and might induce several retinal abnormalities that result in myopia-related visual loss. Thus, it is essential to examine macular layer thicknesses to further investigate myopia-related retinal tissue thinning and stretching and to identify differences in structural features in high myopic eyes compared with normal (control) eyes.

The spectral domain optical coherence tomography (SD-OCT) device enables cross-sectional imaging of the retina. It uses the interference patterns generated by low coherence light reflected by the retina and is a noninvasive tool for quantitative and qualitative measurement of the macula. The latest development of software for segmentation analysis using SD-OCT can allow for easier and more accurate automated differentiation of each retinal layer and measurement of individual layer thickness^17,18^. Several studies have evaluated the effects of age and sex on retinal layer thickness in normal and myopic indivduals^19–22^. Accordingly, we designed this study to focus on structural changes in the thickness of the individual layers of the retina, from the innermost layer (RNFL) to deepest layer (RPE) of the outer retina, between normal axial length and axially elongated highly myopic groups. Considering that the worldwide trend of increasing incidence of myopic patients is relatively greater in Asian countries, our study could provide valuable insight into better understanding the distinguishing characteristics of high myopia compared with control eyes using SD-OCT segmentation analysis.

The primary aim of our study was to investigate and compare the thicknesses of individual retinal layers of the macula between highly myopic eyes and control eyes. We also evaluated the effects of age and sex on thickness of each retinal layer in these groups.

## Results

**Figure 1:**
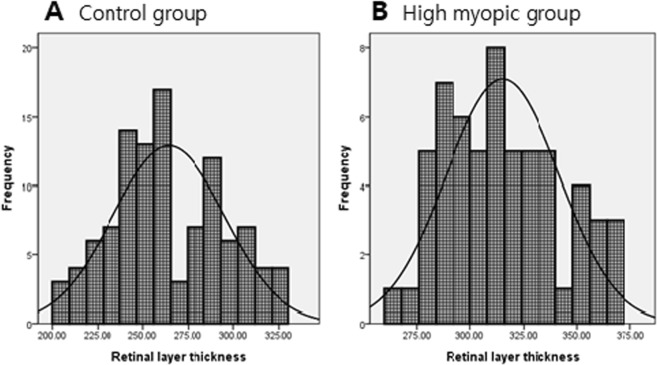
Distribution of total retinal layer thickness (μm) in 105 normal volunteers (**A**) and in 59 high myopia volunteers (**B**) compared with normal distribution (solid curve).

This retrospective review of 378 participants revealed that 164 eyes from 164 individuals met the inclusion and exclusion criteria. The patients were classified into two groups based on AL (26 mm). The high-myopia group (AL ≥ 26 mm) comprised 59 eyes from 33 men and 26 women. The control group (AL < 26 mm) consisted of 105 eyes from 55 men and 50 women. The results for the demographic profiles of the study participants are presented in Table 1. The mean ± standard deviation values for age were 42.92 ± 12.36 years for the high-myopia group and 46.39 ± 18.74 years for the control group. All participants had a Snellen chart BCVA value greater than 20/25. BCVA value was converted into logMAR scale value for statistical analysis; the mean value was logMAR 0.09 ± 0.07 in the high-myopia group and logMAR 0.09 ± 0.06 in the control group (VA range with Snellen chart: 20/16 to 20/25 in both groups). The mean values for SE (diopters) and AL (mm) were −7.33 ± 1.40 and 27.29 ± 0.86 in the high myopia, and −1.22 ± 1.58 and 24.11 ± 1.05 in the control group patients, respectively. There were no between-group differences in mean age, sex, BCVA, and IOP (Table 1). Figure 1 illustrates the frequency distribution of the mean total retina thickness each in normal control and high myopic group, respectively.

**Table 1:** Characteristics of high myopia and control groups.

Variable	Control (AL < 26 mm)	High Myopia (AL ≥ 26 mm)	P-value
Number of subjects (eyes)	105	59	N/A
Age (years)	46.39 ± 18.74	42.92 ± 12.36	0.254
Gender (M/F)	55/50	33/26	0.662^†^
BCVA (logMAR) Visual acuity range (Snellen)	0.09 ± 0.06 20/16 to 20/25	0.09 ± 0.07 20/16 to 20/25	0.765
IOP (mmHg)	16.50 ± 2.55	16.75 ± 2.38	0.815
SE (diopters)	−1.22 ± 1.58	−7.33 ± 1.40	**<0.001**
AL (mm)	24.11 ± 1.05	27.29 ± 0.86	**<0.001**

M, male; F, female; BCVA, best-corrected visual acuity; IOP, intraocular pressure; SE, spherical equivalent; AL, axial length; N/A not applicable.

Values are presented as mean ± standard deviation (count).

P values were yielded by student t-test for comparing continuous values.

^†^P values were yielded by Chi-square statistics for comparing categorical values.

*Bold values indicate statistically significant differences (P < 0.05).

### Comparison of individual retinal layer thicknesses in the macula (5 subfields): high-myopia group (AL ≥ 26 mm) versus the control group (AL < 26 mm)

Table 2 presents the results for the comparison of the thicknesses of individual retinal layers with magnification adjustment in the macula (center subfield; 1-mm foveal zone + superior/nasal/inferior/temporal subfields; 1–3 mm perifoveal zone) between the high myopic eyes and the control eyes. The results for the center subfield analysis prior to adjustment for magnification indicated that the TR, RNFL, GCL, IPL, and OPL thicknesses were significantly greater in patients with an AL longer than 26 mm, compared with those with an AL shorter than 26 mm (P = 0.044, 0.022, 0.004, 0.021, and 0.029, respectively) (see Supplementary Table S1). In the perifoveal region, there were no statistically significant between-group differences in the total retinal thickness of any subfields within 1–3 mm from the fovea. However, there were significant differences for each retinal layer in the perifoveal region: RNFL (superior/nasal/inferior subfields), GCL (all subfields), and IPL (temporal/superior/nasal subfields) thickening with relative thinning of photoreceptors (superior/inferior subfields) and RPE (superior/nasal/inferior subfields) in the high myopic eyes compared with the control eyes (all P < 0.05). After ocular magnification adjustment, all thickness values of the individual retinal layers within the central and entire perifoveal subfields in the high myopic group were significantly thicker than those of control group (all P < 0.05) (Table 2).

**Table 2:** Comparisons of individual retinal layer thickness in the foveal and 4 perifoveal region measured by the automated segmentation of the Spectralis optical coherence tomography after ocular magnification adjustment.

Thickness Parameter	All Participants (N = 164)	Control Group (AL < 26 mm; N = 105)	High Myopic Group (AL ≥ 26 mm; N = 59)	P-value*
**Total retina**				
Center subfield	282.4 ± 38.2	264.3 ± 30.9	315.2 ± 26.5	**<0.001**
Temporal subfield	339.9 ± 35.3	321.2 ± 26.7	373.8 ± 20.8	**<0.001**
Superior subfield	352.6 ± 36.5	333.4 ± 27.8	387.5 ± 21.2	**<0.001**
Nasal subfield	355.2 ± 38.5	335.5 ± 29.3	390.7 ± 25.3	**<0.001**
Inferior subfield	348.7 ± 37.4	329.6 ± 30.2	383.2 ± 20.7	**<0.001**
**Retinal nerve fiber layer**				
Center subfield	12.3 ± 2.9	11.3 ± 2.8	14.3 ± 2.1	**<0.001**
Temporal subfield	18.3 ± 2.3	17.3 ± 1.7	20.1 ± 2.3	**<0.001**
Superior subfield	26.7 ± 5.8	24.1 ± 4.7	31.4 ± 4.5	**<0.001**
Nasal subfield	22.8 ± 4.8	20.9 ± 3.7	26.4 ± 4.5	**<0.001**
Inferior subfield	26.7 ± 6.1	24.0 ± 4.5	31.5 ± 5.7	**<0.001**
**Ganglion cell layer**				
Center subfield	15.9 ± 5.7	14.0 ± 5.4	19.3 ± 4.7	**<0.001**
Temporal subfield	48.2 ± 9.8	43.8 ± 8.2	56.1 ± 7.1	**<0.001**
Superior subfield	53.5 ± 8.6	49.8 ± 7.5	60.2 ± 6.2	**<0.001**
Nasal subfield	52.4 ± 10.1	48.2 ± 9.0	60.2 ± 6.9	**<0.001**
Inferior subfield	52.3 ± 9.7	48.4 ± 9.3	59.2 ± 5.6	**<0.001**
**Inner plexiform layer**				
Center subfield	21.5 ± 4.8	19.6 ± 4.3	24.8 ± 3.9	**<0.001**
Temporal subfield	42.2 ± 6.5	39.2 ± 5.3	47.7 ± 4.6	**<0.001**
Superior subfield	41.9 ± 5.8	39.2 ± 4.8	46.7 ± 4.2	**<0.001**
Nasal subfield	43.6 ± 6.9	40.3 ± 5.4	49.6 ± 4.8	**<0.001**
Inferior subfield	41.6 ± 6.2	39.0 ± 5.5	46.4 ± 4.2	**<0.001**
**Inner nuclear layer**				
Center subfield	20.8 ± 6.2	19.3 ± 6.5	23.6 ± 4.7	**<0.001**
Temporal subfield	39.2 ± 4.9	37.4 ± 4.6	42.5 ± 3.8	**<0.001**
Superior subfield	42.8 ± 4.7	41.1 ± 4.6	45.8 ± 3.1	**<0.001**
Nasal subfield	43.0 ± 5.4	41.1 ± 5.0	46.4 ± 4.1	**<0.001**
Inferior subfield	42.8 ± 5.7	40.8 ± 5.2	46.6 ± 4.6	**<0.001**
**Outer plexiform layer**				
Center subfield	28.8 ± 9.2	26.1 ± 7.5	33.8 ± 9.9	**<0.001**
Temporal subfield	35.9 ± 10.5	33.1 ± 9.5	41.0 ± 10.3	**<0.001**
Superior subfield	39.9 ± 12.6	37.6 ± 11.5	44.2 ± 13.4	**0.001**
Nasal subfield	38.6 ± 12.2	36.9 ± 10.8	41.7 ± 14.0	**0.014**
Inferior subfield	39.8 ± 12.0	37.1 ± 10.6	44.8 ± 12.8	**<0.001**
**Outer nuclear layer**				
Center subfield	92.4 ± 15.9	87.2 ± 15.3	101.9 ± 12.3	**<0.001**
Temporal subfield	71.0 ± 11.9	68.3 ± 11.5	75.7 ± 11.1	**<0.001**
Superior subfield	64.0 ± 14.5	61.1 ± 13.6	69.1 ± 14.8	**<0.001**
Nasal subfield	69.2 ± 16.7	66.0 ± 15.3	75.1 ± 17.6	**<0.001**
Inferior subfield	61.7 ± 14.3	60.0 ± 13.7	64.6 ± 15.0	**<0.001**
**Photoreceptors**				
Center subfield	74.4 ± 6.7	71.4 ± 5.9	79.9 ± 4.0	**<0.001**
Temporal subfield	70.1 ± 5.7	67.2 ± 4.5	75.5 ± 2.9	**<0.001**
Superior subfield	68.4 ± 5.4	65.5 ± 4.2	73.6 ± 2.6	**<0.001**
Nasal subfield	69.2 ± 5.7	66.3 ± 4.5	74.5 ± 3.6	**<0.001**
Inferior subfield	68.5 ± 5.5	65.5 ± 4.4	73.9 ± 2.3	**<0.001**
**Retinal pigment epithelium**				
Center subfield	17.0 ± 2.8	16.2 ± 2.5	18.6 ± 2.6	**<0.001**
Temporal subfield	14.9 ± 1.8	14.4 ± 1.8	15.9 ± 1.4	**<0.001**
Superior subfield	15.9 ± 2.1	15.4 ± 2.2	16.7 ± 1.6	**<0.001**
Nasal subfield	15.8 ± 2.2	15.3 ± 2.1	16.7 ± 2.1	**<0.001**
Inferior subfield	15.2 ± 2.1	14.8 ± 2.2	16.0 ± 1.6	**<0.001**

AL, axial length.

Values are presented as mean thickness ± standard deviation; SD (µm).

P values were yielded by independent student t-test between the control group (AL < 26 mm) and high myopia group (AL ≥ 26 mm).

*Bold values indicate statistically significant differences (P < 0.05).

### Effect of sex on individual retinal layer thicknesses in different axial length group

In the control group (AL < 26 mm), all the retinal layers of the central macula were significantly thicker in men compared with women (all P < 0.05), except for the OPL after the correction of magnification error (OPL, photoreceptors, and RPE before magnification adjustment; see Supplementary Table S2). In the high myopic patients (AL ≥26 mm), there were no significant sex-dependent thickness differences for any of the retinal layers, except for RPE thickness regardless of magnification adjustment (P = 0.001) (Tables 3 and S2).

**Table 3:** Effect of sex on individual retinal layer thicknesses and axial length prolongation after ocular magnification adjustment.

Control Group (AL < 26 mm)	Male (n = 55)	Female (n = 50)	Mean difference	P-value*
Total retina	276.9 ± 26.8	245.6 ± 26.9	31.3 ± 5.3	**<0.001**
Retinal nerve fiber layer	12.6 ± 2.3	9.4 ± 2.3	3.2 ± 0.5	**<0.001**
Ganglion cell layer	15.2 ± 4.8	12.2 ± 5.7	3.0 ± 1.0	**0.004**
Inner plexiform layer	21.2 ± 4.0	17.2 ± 3.7	3.9 ± 0.8	**<0.001**
Inner nuclear layer	21.1 ± 6.1	16.6 ± 6.0	4.5 ± 1.2	**<0.001**
Outer plexiform layer	27.1 ± 7.8	24.5 ± 6.9	2.6 ± 1.5	0.075
Outer nuclear layer	90.6 ± 17.4	82.2 ± 9.5	8.4 ± 2.9	**0.005**
Photoreceptors	72.4 ± 5.2	69.9 ± 6.7	2.4 ± 1.1	**0.036**
Retinal pigment epithelium	16.6 ± 2.0	15.5 ± 3.0	1.1 ± 0.5	**0.028**
**High Myopic Group (AL ≥ 26 mm)**	**Male (n = 33)**	**Female (n = 26)**	**Mean difference**	P-value*
Total retina	318.4 ± 27.5	303.7 ± 19.2	14.7 ± 8.2	0.077
Retinal nerve fiber layer	14.3 ± 2.0	14.2 ± 2.6	0.1 ± 0.7	0.859
Ganglion cell layer	19.8 ± 4.8	17.5 ± 3.9	2.4 ± 1.5	0.107
Inner plexiform layer	25.3 ± 4.0	23.3 ± 3.2	1.9 ± 1.2	0.115
Inner nuclear layer	23.8 ± 4.7	23.1 ± 4.9	0.7 ± 1.5	0.616
Outer plexiform layer	34.8 ± 10.3	30.2 ± 7.6	4.6 ± 3.1	0.136
Outer nuclear layer	102.1 ± 12.0	100.9 ± 13.8	1.2 ± 3.9	0.762
Photoreceptors	80.2 ± 3.7	78.8 ± 5.0	1.4 ± 1.3	0.259
Retinal pigment epithelium	19.2 ± 2.4	16.5 ± 2.3	2.7 ± 0.8	**0.001**

AL, axial length.

Values are presented as mean thickness ± standard deviation (µm).

P values were yielded by student t-test between male and female in each group divided by AL.

*Bold values indicate statistically significant differences (P < 0.05).

### Effect of age on individual retinal layer thicknesses in different axial length group

After adjusting for sex, there was a partial correlation between age and the thickness of each retinal layer in the center and four perifoveal subfields within the 3-mm ETDRS ring in each group after magnification correction (Table 4). In subjects with an AL < 26 mm, the thicknesses of the total retina (all subfields), RNFL (superior/inferior subfields), GCL (all subfields except center), IPL (all subfields except center), INL (superior subfield), OPL (center/superior/temporal subfields), photoreceptors (all subfields), and RPE (center subfield) decreased significantly with increasing age (all P < 0.05). In patients with an AL ≥ 26 mm, only a few retinal layers (the GCL of the inferior subfield, IPL of nasal/inferior subfields, INL of nasal subfield, and RPE of the center subfield) showed a significant correlation with increasing age (all P < 0.05) (Table 4). We also showed the effect of age on individual thickness without magnification adjustment in Supplementary Table S3.

**Table 4:** Effect of age on individual retinal layer thicknesses and axial length prolongation after ocular magnification adjustment by correlation analysis.

Thickness parameter	Control Eyes (AL < 26 mm)		High Myopic Eyes (AL ≥ 26 mm)	
	r	P*	r	P*
**Total retina**				
Center subfield	−0.270	**0.005**	0.087	0.518
Temporal subfield	−0.448	**<0.001**	−0.079	0.556
Superior subfield	−0.484	**<0.001**	−0.083	0.535
Nasal subfield	−0.399	**<0.001**	−0.049	0.715
Inferior subfield	−0.452	**<0.001**	−0.182	0.171
**Retinal nerve fiber layer**				
Center subfield	−0.062	0.530	0.057	0.673
Temporal subfield	0.055	0.573	−0.183	0.170
Superior subfield	−0.222	**0.022**	0.215	0.106
Nasal subfield	−0.180	0.065	0.065	0.628
Inferior subfield	−0.280	**0.004**	−0.087	0.515
**Ganglion cell layer**				
Center subfield	−0.175	0.072	0.039	0.774
Temporal subfield	−0.540	**<0.001**	−0.102	0.447
Superior subfield	−0.536	**<0.001**	0.038	0.779
Nasal subfield	−0.478	**<0.001**	−0.055	0.683
Inferior subfield	−0.506	**<0.001**	−0.312	**0.017**
**Inner plexiform layer**				
Center subfield	−0.182	0.062	−0.050	0.709
Temporal subfield	−0.527	**<0.001**	−0.160	0.232
Superior subfield	−0.547	**<0.001**	−0.126	0.347
Nasal subfield	−0.466	**<0.001**	−0.324	**0.013**
Inferior subfield	−0.491	**<0.001**	−0.383	**0.003**
**Inner nuclear layer**				
Center subfield	0.085	0.387	0.233	0.078
Temporal subfield	−0.160	0.101	−0.176	0.187
Superior subfield	−0.195	**0.045**	−0.109	0.415
Nasal subfield	−0.033	0.740	0.319	**0.015**
Inferior subfield	−0.074	0.454	0.075	0.575
**Outer plexiform layer**				
Center subfield	−0.196	**0.044**	0.004	0.976
Temporal subfield	−0.224	**0.021**	0.205	0.123
Superior subfield	−0.215	**0.027**	−0.020	0.882
Nasal subfield	−0.070	0.473	−0.036	0.790
Inferior subfield	−0.048	0.622	0.130	0.331
**Outer nuclear layer**				
Center subfield	−0.050	0.613	0.197	0.139
Temporal subfield	−0.018	0.857	−0.025	0.851
Superior subfield	0.036	0.713	−0.105	0.432
Nasal subfield	−0.066	0.501	0.045	0.736
Inferior subfield	−0.112	0.254	−0.088	0.510
**Photoreceptors**				
Center subfield	−0.334	**<0.001**	−0.041	0.763
Temporal subfield	−0.334	**<0.001**	−0.184	0.166
Superior subfield	−0.475	**<0.001**	−0.179	0.179
Nasal subfield	−0.426	**<0.001**	−0.107	0.426
Inferior subfield	−0.417	**<0.001**	−0.251	0.057
**Retinal pigment epithelium**				
Center subfield	−0.259	**0.007**	−0.427	**0.001**
Temporal subfield	−0.046	0.638	−0.227	0.086
Superior subfield	−0.007	0.943	−0.091	0.495
Nasal subfield	−0.032	0.748	−0.252	0.057
Inferior subfield	−0.074	0.453	−0.139	0.299

AL, axial length.

P values were yielded by partial correlation analysis (Pearson correlation) after adjusting for sex to evaluate the effect of age on macular thickness in each other group (r = partial correlation coefficients).

*Bold values indicate statistically significant differences (P < 0.05).

### Effect of axial length on individual retinal layer thickness in central macula after ocular magnification adjustment

The relationships between axial length and central individual retinal layer thicknesses are shown in Table 5. All central macular OCT parameters with correction of magnification error showed significant positive correlations with axial length. Also, without magnification adjustment, as seen in Supplementary Table S4, we noted statistically significant positive correlations only in the thicknesses of the total retina, inner retina (RNFL, GCL, IPL, INL), and OPL.

**Table 5:** Correlation of the axial length with the thicknesses obtained by the OCT after adjusting for the ocular magnification.

Center subfield	r	95% CI	P
Total retina	0.786	0.720 to 0.838	**<0.001**
Retinal nerve fiber layer	0.541	0.424 to 0.641	**<0.001**
Ganglion cell layer	0.536	0.418 to 0.637	**<0.001**
Inner plexiform layer	0.629	0.527 to 0.713	**<0.001**
Inner nuclear layer	0.440	0.308 to 0.555	**<0.001**
Outer plexiform layer	0.516	0.394 to 0.619	**<0.001**
Outer nuclear layer	0.476	0.349 to 0.586	**<0.001**
Photoreceptors	0.796	0.732 to 0.846	**<0.001**
Retinal pigment epithelium	0.555	0.440 to 0.652	**<0.001**

CI, confidential interval.

P values were yielded by Pearson correlation analysis to evaluate the effect of axial length on macular thickness (r = correlation coefficients).

*Bold values indicate statistically significant differences (P < 0.05).

## Discussion

This study was performed to compare the individual retinal layer thicknesses of the macula between non-pathologic high myopic patients and control subjects using segmentation analysis of SD-OCT images. We found that axially elongated high myopic eyes (AL ≥ 26 mm) had different structural features compared with control eyes (AL < 26 mm), with significantly greater individual macular layer thicknesses that were independent of sex and age.

High myopia is defined as an SE < −6 diopters or an AL ≥ 26 mm^4–6^. We chose to use the latter criteria as the standard of classification into the two groups (i.e., high-myopia group and control group). Most previous studies adjusted the collected data for well-known confounders, such as sex and SE^23–28^. A recent study by Liu *et al*. also investigated intraretinal layer profiles within 6-mm diameter circle in emmetropic, mild, and high myopic eyes using ultrahigh-resolution OCT^19^. However, adjusting for SE might have introduced errors in this analysis because refractive SE can vary under certain conditions (e.g., cataract progression, presence of macula disease, post-refractive surgery). On the other hand, AL is a relatively constant parameter during adulthood, after eyeball growth is completed at approximately 20 years of age^4^. Hence, our study classified study groups based on AL, and further evaluated the effects of age and sex on segmented layer thicknesses between groups.

Due to ocular magnification of individual optical systems, the retinal thickness measured from a specific area can vary with different axial length among individuals. To obtain an accurate measurement of actual thickness and minimize magnification errors during analysis, obtained thickness values should be corrected according to several methods described in previous studies (e.g., Littmann’s method, Bennett formula, and Kang’s method)^29–31^. With ocular magnification adjustment, we can calculate the actual average thickness of all individual retinal layers and evaluate the genuine structural features in axially elongated high myopic patients, compared with normal axial length individuals.

The topographical variation in the high myopic patients’ retinal thicknesses was different from those of the control subjects (Table 2). The results of our study before magnification adjustment indicated that the total retinal thickness of the central subfield was significantly greater in the high myopic group with the longer AL compared with the control group with less than 26-mm AL (P < 0.05). The results for the analysis of the individual layers of the macula indicated that similar topographical variations with greater thickness were present in the RNFL, GCL, IPL, and OPL. With regard to the retinal thickness of the perifoveal area (1–3 mm from the fovea), we did not find significant between-group differences in total thickness. Whereas, thickening of the RNFL (superior/nasal/inferior subfields), GCL (all subfields), and IPL (temporal/superior/nasal subfields) with thinning of the photoreceptors (superior/inferior subfields) and RPE (superior/nasal/inferior subfields) were significantly associated with a longer AL (all P < 0.05). However, the thickness values of all individual retinal layers within the entire macula region in the axially elongated group were significantly thicker than those of control group after adjustment for ocular magnification (all P < 0.05). These findings have two interesting clinical implications. First, there was an apparent tendency for greater thickness of the macula in the high myopia group. Second, this tendency towards a thicker retina was mostly attributable to the greater thickness of the inner retina in high myopia patients.

A prior study reported macular thickness profiles of intraretinal layers in myopia using OCT^19^. Results from that study showed that the total thickness of the central region in the high myopic group did not have significant difference with other groups (e.g., emmetropia, mild to moderate myopia); our results, on the other hand, showed significant thickening of the total retina in the high myopic group. With the exception of the inner retina layers, which were not included in the analysis of the central region in the previous study, the outer retinal layer’s results were largely inconsistent with our results after magnification adjustment regarding the central zone in high myopia: in the perifoveal area, significant thickening of the RNFL, OPL, and photoreceptor in high myopia was found in both studies, while, contrary to our results, thinning of the INL and ONL in high myopia was reported. We believe that such differences may have resulted from the fact that the previous study used “spherical equivalent” to divide the study groups in terms of degree of myopia. Also, participants enrolled in the previous study were very young (mean age in their 20 s), and mean axial length of the high myopic group was 26.6 mm which is a very mild form of high myopia. On the other hand, the grouping criteria of our study used “axial length” as a fixed factor, and the mean age of our patients was in their 40 s while the mean axial length of the high myopia group without pathologic findings was 1 mm longer than in the study by Liu *et al*. implying that our results might reflect more ocular magnification effect.

Many previous investigations have revealed that an axial elongation is mainly responsible for myopic enlargement of the globe (i.e., rather than changes in horizontal and vertical globe diameters). It is associated with thinning of the retina in the equatorial and pre-equatorial regions; the foveal region is less affected^32^. Other studies have revealed that foveal retinal thickness does not decrease with a longer AL and that the BCVA of eyes with no myopic retinopathy is independent of AL^23,32,33^. Duan *et al*. found that central foveal thickness has a weak positive correlation with AL in adults with normal axial length^23^. Wu *et al*. found that mean foveal thickness of non-myopic eyes is significantly thinner than that of high myopic eyes^33^. The results of these studies are consistent with our study in that although a significant difference was present only in the central subfield, high myopia tended to increase in the total retinal thickness of the macula. Our study results did somewhat contrast with the results of previous studies in that although not statistically significant, high myopia patients also had perifoveal full-layer thickening with increasing thickness of the inner retina and decreased outer retinal thickness. Partially contrary to our results, another study found that the mean foveal thickness increased, but inner/outer macular ring thickness decreased, with a longer AL^34^. The equivalent or thicker tendency of the macula layer in the high-myopia group (AL ≥ 26 mm) compared with the control group (AL < 26 mm) may be explained by recent findings that myopic axial elongation was not associated with a stretching and lengthening of the macular region of the Bruch’s membrane^35^.

A recent study investigated correlations between peripapillary retinal nerve fiber layer thickness and axial length in healthy eyes^20^. They demonstrated that uncorrected average RNFL thickness decreases with axial elongation before magnification adjustment, while after correction of average RNFL thickness with ocular magnification adjustment, there was a weak positive correlation with increasing axial length, emphasizing the effect of ocular magnification on RNFL measurement. Our study showed that the statistically significant thickening of the central area of the total retina and individual layers of the inner retina (e.g., RNFL, GCL, and IPL) in the central and most of the perifoveal zones, as well as some of the perifoveal RPE, in highly myopic patients, compared with normal individuals, prior to adjustment for magnification. However, significant thickening of all individual retinal layers, including the total, inner, and outer retina, within the entire macula in axially elongated patients was noted after correction of magnification error. Moreover, they reported the effects of refractive errors and magnification factors, and the mean scan circle magnification for the myopic eye of −4D or less was >5%. Therefore, they recommended that ocular magnification adjustment should be considered when exceeding −4D of myopia. According to their results, the highly myopic group < −6.0D, with a mean axial length of 26.82 mm, had a magnification effect of an average of 1.1043, compared with 0.9869 of low diopter (−0.50 to −1.99D) myopia, with a mean AL of 24.16 mm. Our study showed similar adjustment effects in an axial elongated group (mean AL: 27.29 mm), compared to a control group (mean AL: 24.11 mm), after correction for ocular magnification error.

Even given these findings, it still remains unclear why axially elongated high myopia was significantly associated with increased macular thickness and why the thickening of individual layer was predominantly in the inner retina rather than the outer retina even when without adjustment for magnification effect as well as with correction. The individual macular layers where thickening occurred in the high myopia patients should be noted. These layers included the RNFL, GCL, IPL, and OPL, which mostly consist of neuronal synapses. The layers did not include the INL, ONL, and photoreceptors, which mostly consist of neuronal cell bodies. We hypothesize that in axially elongated eyes, as myopic enlargement of the eyeball proceeded axially before adulthood, a myopic degree of refractive error was aggravated. This increase resulted in blurred vision and progressive visual loss. Therefore, we speculate that as part of an effort to obtain clear visual images, a process such as *“reinforcement in retinal neuronal synapses”* can occur in the retina of individuals with axial elongated high myopia^36^. To transmit a greater amount of, and clear, visual information to brain using a relatively constant number of retinal neuronal cells, the dendrites or branched foot plates of neuronal cell bodies could be activated or hypertrophied at the synaptic connections corresponding the OPL, IPL, and GCL. This compensatory change might be associated with an increase in thickness of each corresponding retinal layer. And another assumption is that the plexiform layers are thicker with axial prolongation due to axial pulling forces on the retina, which might be a precursor to retinoschisis found in progressive pathologic myopia. Our results revealed a tendency towards significant thickening of the corresponding retinal layers (RNFL, GCL, IPL, and OPL of the center subfields and most subfields of the perifoveal area) in the elongated high myopic eyes. Accordingly, we carefully could hypothesize that these microstructural changes of highly myopic eyes in our study might represent an intermediate stage during progression towards development of pathological myopia, before constant axial elongation and extensive retinal thinning; however, this is only speculative and needs further investigation in a prospective study in the future.

Except for the RPE layer, we did not find between-sex differences in individual retinal layers in the central macula of high myopic eyes. On the contrary, in the control eye group, men had thicker retinal layers than women. This difference included the total retina and all individual retinal layers, except OPL. This finding was partially consistent with a previous study that revealed that the full thickness and inner retina thickness of the central subfield in female subjects are lower than in male subjects and that there are no ethnicity-, age-, or sex-related differences in the outer retina^37^. Wang *et al*. found that the central subfield thickness is higher for men than for women, but that there is no association with AL^38^. The difference between the results might be due to the presence of different study population characteristics. Wang *et al*. included patients with AL values ranging from 20.9 mm to 26.9 mm, but we separated the two groups using an AL value of 26 mm. Our results indicated that control eyes with an AL < 26 mm had similar characteristics to those found by Wang *et al*. The reason that RPE thickening in men was the only sex-associated difference in the high myopic eyes examined in this study remains unclear. Further investigations to identify and confirm the cause of this finding are necessary.

Our study also revealed that individual retinal thickness in the macula was negatively correlated with age in eyes with an AL < 26 mm, predominantly in the inner retina, photoreceptor, and total retina. This finding of age-related inner retina thinning compares to the finding of other studies that aging is associated with loss of retinal neurons and glial cells^22,37–41^. In contrast, there were only a few retina layers of significant correlations with aging, including some part of perifoveal GCL, IPL, INL and the central RPE in the high myopia patients. This result suggested that elongated eyes with an AL ≥ 26 mm seemed to be less affected by aging (except for the RPE layer). This result may be explained by the effects of the process of reinforcement of neuronal synapses, which could aid in the development of relatively compact and strong retinal tissue that is more resistant to aging process. However, these results have a limitation in that simple change of each retinal thicknesses as age increases could not directly reflect the age-related retinal cell death, so further study on retinal histopathologic changes with thickness by aging process will be needed.

Some previous study results indicated that the increased foveal thickness of myopic patients could be due to the poorer foveal fixation that may be present when high myopia is present. This change causes overestimation when thickness is measured and is due to an optical artifact, not an anatomical change^37,42^. However, we speculate that our findings of macular thickness in high myopia was more likely associated with axial elongation than a simple effect of error. The recently developed and introduced Spectralis OCT enabled us to obtain high-definition OCT images of the macula with higher reliability and reproducibility using eye-tracking software (TruTrack, Heidelberg Engineering, Inc.). Also, another study demonstrated that refractive error, especially a higher degree of myopia, was significantly associated with presence of segmentation errors defined as mislocated lines between retinal layers when assessing macular GCL-IPL thickness using OCT^43^. To minimize these segmentation errors of high myopia, we confirmed that automated lines were in proper position between retinal layers and there were no presence of obvious myopic tilted images through repetitive measurements. Thus, we think that measurement error was unlikely to affect our findings.

Some reports demonstrated a change in correlation from negative to positive between average RNFL thickness and axial length after adjustment using Littman’s method^20,22^. Actually, questions regarding the use of Littman’s method to adjust OCT parameters as a depth unit have been raised, suggesting that it might not be suitable for the correction of tissue thickness through magnification effects based on a few reasons: Measured tissue thickness in OCT is a distance with a z-axis direction, unlike the fundus photography, a simple multiplication formula cannot be used to correct tissue thickness. Also, Littmann’s method may not be applied to nontelecentric systems of OCT instruments^22^. Considering this point of view, we decided to present the results of our study before (see Supplementary Tables S1–S4) and after correction (Tables 2–5) of ocular magnification error. Regardless of ocular magnification adjustment, our results consistently showed distinctive structural differences in the axially elongated high myopic group, compared with the normal controls, including the tendency for macular thickening predominantly in the inner retina, fewer gender differences, and less sensitivity to aging.

This study had some limitations. We used a retrospective study design that included cross-sectional rather than longitudinal analysis of the data. Therefore, we could not evaluate longitudinal changes in retinal thickness associated with the aging process over time within the same individual. We investigated the microstructural features of the retinal layer in non-pathologic healthy Korean people with a high incidence of myopia; therefore, ethnic group differences should be considered when interpreting results in comparison to those from prior studies. Also, we were not able to correlate the structural features in our results with visual functions, using methods such as multifocal electroretinography. To obtain good quality SD-OCT data, our study population only included patients without ophthalmic disease. Therefore, it is possible that the results might have been affected by selection bias. Additionally, we could not fully analyze the individual retinal thickness of 6-mm grid of ETDRS ring because of OCT scanning mode in our clinic, and the thickness results of 6-mm zone might help clarify the trends of our results. Additional well-designed prospective studies that include a greater number of subjects are required to confirm and complement our findings.

In conclusion, this study revealed that axially elongated, highly myopic eyes had individual retinal thickness characteristics that were structurally distinguishable from non-elongated eyes: 1) there was a tendency towards macular thickening, predominantly in the inner retina, 2) there were no sex-related differences, and 3) the changes were resistant to the aging process. These findings should be considered during interpretation of segmentation data from high myopia patients with an elongated AL.

## Methods

This study was performed as a retrospective, non-interventional comparative analysis. The protocol was approved by the Institutional Review and Ethics Boards of the Yonsei University, Gangnam Severance Hospital, which provided a waiver of informed consent for the retrospective review of existing patient records (IRB approval number: 3-2015-0311). It was performed in accordance with the tenets of the Declaration of Helsinki. The medical records and imaging data of 378 eyes from 378 patients over the 2012–2016 period were retrospectively reviewed. No patients had previous ocular surgery at our institution. Age, sex, and other relevant medical history information was obtained and patients whose eyes met the inclusion and exclusion criteria were selected. All patients who visited our retina clinic of ophthalmology for retinal examination on the day of presentation underwent a comprehensive ophthalmologic examination, which included measurement of best-corrected visual acuity (BCVA) assessed using Snellen chart, intraocular pressure (IOP) measurement by non-contact tonometer, slit-lamp biomicroscopy, dilated fundus examination using indirect ophthalmoscopy, refraction measurement with automated keratometry (KR-1, Topcon, Tokyo, Japan) for calculation of spherical equivalent, axial length measurement by IOLMaster^®^ instrument (Carl Zeiss Meditec, Inc., Jena, Germany), and macular layer segmentation analysis using SD-OCT device (Spectralis OCT Family Acquisition Module, version 6.3.4.0, Heidelberg Engineering, Inc., Heidelberg, Germany).

The inclusion criteria were as follows: no diagnosis of any kind of previous or current ocular disease; no systemic underlying disease (e.g., hypertension, diabetes) or any other infectious or autoimmune diseases that could affect retinal structure; corrected visual acuity greater than 20/25 measured using a Snellen chart; a normal range in IOP (between 10 mmHg and 21 mmHg); an available SD-OCT examination result (signal strength >25 within a range of 0–40); and absence of definite artifacts or one or more missing areas. Subjects were excluded based on any of the following criteria: eyes with any previous or current conditions affecting the macula, optic nerve (e.g., diabetic retinopathy, uveitis, macular hole, epiretinal membrane, glaucoma, or optic nerve abnormalities), or the presence of structural abnormalities, including myopic pathologic changes such as myopic retinoschisis, myopic macular hole (partial or complete), posterior staphyloma, and myopic choroidal neovascularization signs observed on OCT images; corrected visual acuity less than 20/25 or abnormal IOP range; previous history of ocular trauma, ocular surgery, or current use of medications that could affect the retinal structure or visual function. The recruited subjects that met the criteria were divided into two groups using a 26-mm AL cut-point (i.e., high-myopia group (AL ≥ 26 mm), non-high myopia group (AL < 26 mm))^4^. Among the two eyes, one eye of each patient was randomly selected for analysis.

**Figure 2:**
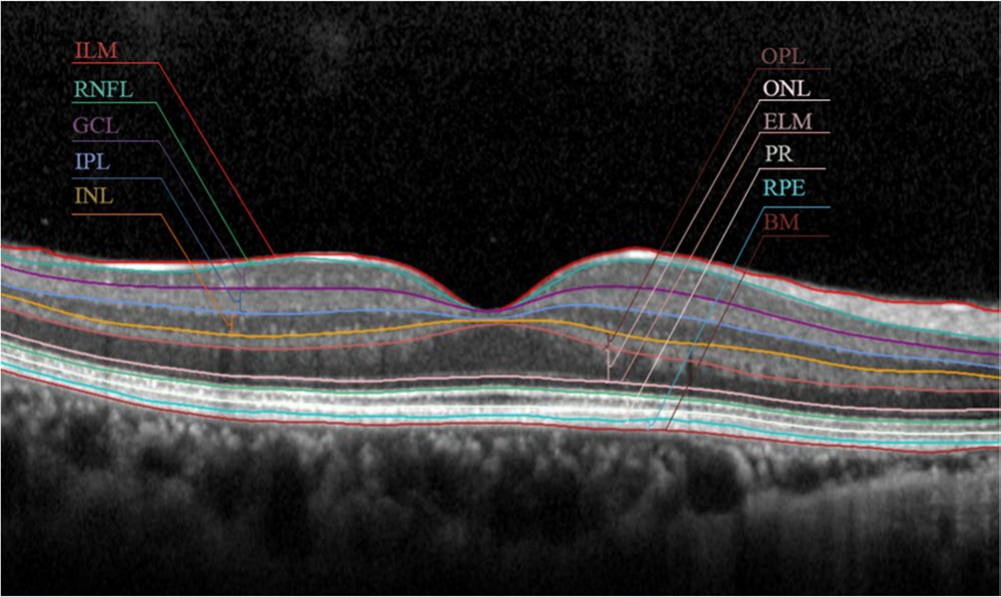
A representative figure of retinal layer division determined by the automated segmentation application of the Spectralis optical coherence tomography (OCT). The segmentation software automatically marked the 10 retinal layers (ILM = inner limiting membrane; RNFL = retinal nerve fiber layer; GCL = ganglion cell layer; IPL = inner plexiform layer; INL = inner nuclear layer; OPL = outer plexiform layer; ONL = outer nuclear layer; ELM = external limiting membrane; PR = photoreceptors; RPE = retinal pigment epithelium; BM = Bruch’s membrane).

All eyes included in this study underwent SD-OCT scanning of the macular area after pupil dilation. Each scan was performed by the same experienced operator. OCT images were acquired by obtaining perifoveal volumetric retinal scans comprising 37 A-scans with 496 pixels in depth and 768 pixels in width (scanning area: 6 mm × 3 mm, centered on the fovea). The automated retinal segmentation of SD-OCT images (Segmentation Technology, Heidelberg Engineering, Inc.) allowed automatic segmentation of the retinal layers in a single horizontal scan of the foveal area. The retinal layer was separated into 10 layers and the mean thickness of each retinal layer was measured (Fig. 2).

Our study evaluated and compared thickness values between different axial length groups, and we performed additional analysis after adjusting for the thickness of individual retinal layers for ocular magnification effects based on axial length. The thickness values for the individual retinal layers were analyzed and used as the main outcomes to evaluate the distinctive structural features of highly myopic eyes relative to control eyes.

**Figure 3:**
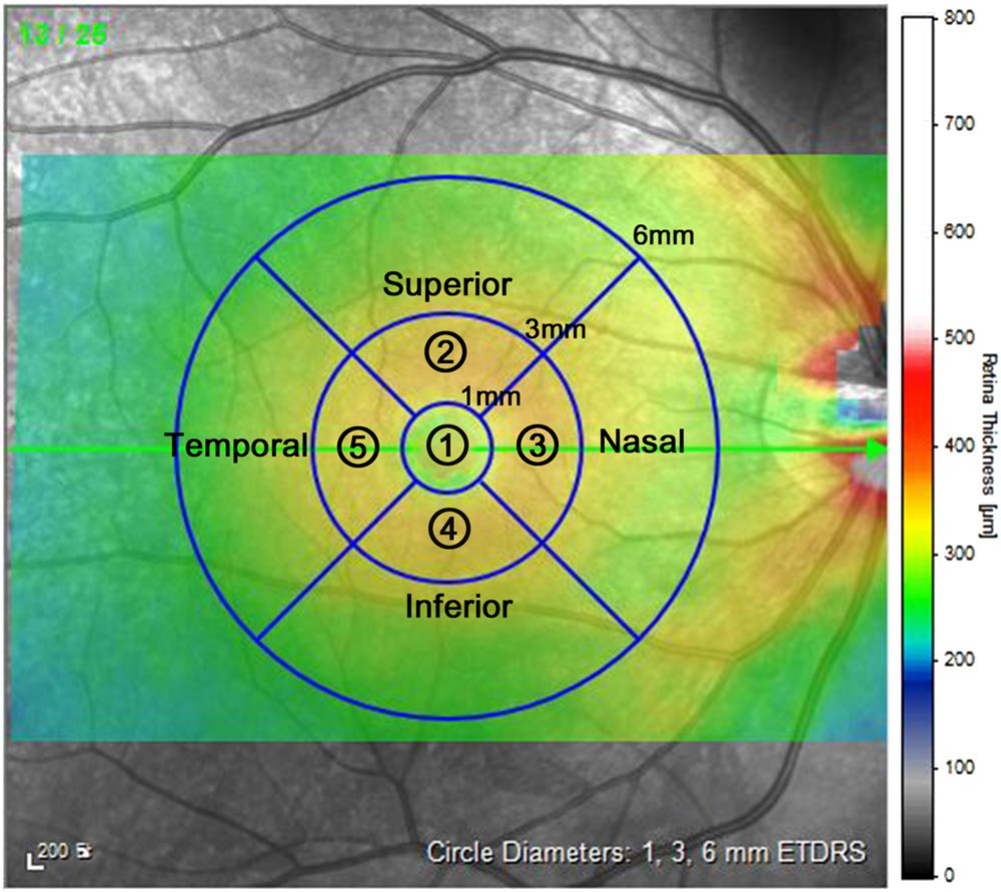
Mean individual retinal layer thickness measurement in the 5 subfields of the ETDRS region. Foveal zone; within 1 mm radius ETDRS circle (region ①) and the 4 perifoveal regions around the center fovea; composed of superior, nasal, inferior and temporal subfields within 1–3 mm radius ETDRS circle (region ②–⑤, respectively).

After automated segmentation of the retinal layer, the Spectralis mapping software generated automated measurements of the thicknesses of individual retinal layers on the central 1-mm, 3-mm, and 6-mm subfields as defined by the Early Treatment Diabetic Retinopathy Study (ETDRS). Among those regions of the ETDRS ring, we analyzed the individual retina layer thickness in a total five subfields corresponding to the macula: the center subfield (foveal area within central 1-mm zone) and the superior/nasal/inferior/temporal subfields (four perifoveal areas within 1–3 mm zone from the fovea) (Fig. 3).

### Statistical analyses

The statistical analysis was performed using SPSS software (version 21.0, SPSS Inc., Chicago, IL, USA). The independent Student’s t-test and Pearson’s chi-square test were used to compare demographic characteristics between groups. The t-test was used to compare the mean value for each retina layer thickness after ocular magnification adjustment between the high myopic group and the control group and between males and females. The partial correlation test was used to determine the effect of age on individual retina layers while controlling for potential confounding variables, such as sex and the axial length on the OCT parameters presenting correlation coefficient as well as 95% confidence interval. A P-value < 0.05 was considered to indicate a statistically significant result.

## Supplementary information

Supplementary information files

## Data Availability

The datasets used and/or analyzed during the current study from the corresponding author on reasonable request.
