# Supplementary material for: Comparison of Individual Retinal Layer Thicknesses between Highly Myopic Eyes and Normal Control Eyes Using Retinal Layer Segmentation Analysis
Source: Sci Rep. 2019 Sep 30;9:14000. doi: 10.1038/s41598-019-50306-w (PMC6769047; doi:10.1038/s41598-019-50306-w)
Supplement: Supplementary file 1 — Supplementary information files [file 41598_2019_50306_MOESM1_ESM.pdf]

Comparison of Individual Retinal Layer Thicknesses between Highly Myopic Eyes and Normal Control Eyes Using Retinal Layer Segmentation Analysis

Jin Hyung Kim[1,2†], Sung Hoon Lee[3†], Jae Yong Han[3], Hyun Goo Kang[3], Suk Ho Byeon[1], Sung Soo Kim[1], Hyoung Jun Koh[1], Sung Chul Lee[1] & Min Kim[3*]

[†]These authors contributed equally to this work.

[1]Institute of Vision Research, Department of Ophthalmology, Severance Hospital, Yonsei University College of Medicine, 134 Shinchon-dong, Seodaemun-gu, Seoul, Korea

[2]Eyereum Eye Clinic, Seoul, Korea

[3]Department of Ophthalmology, Gangnam Severance Hospital, Yonsei University College of Medicine, 211, Eonjuro, Gangnam-gu, Seoul, Korea

Corresponding author:

Min Kim, M.D.-Ph.D.
Department of Ophthalmology, Institute of Vision Research, Gangnam Severance Hospital, Yonsei University College of Medicine
211, Eonjuro, Gangnam-gu, Seoul, Korea, 135-270
Phone: 82-2-2019-3440
Fax: 82-2-3463-1049
E-mail: minkim76@gmail.com

Table S1. Comparisons of individual retinal layer thickness in the foveal and 4 perifoveal region measured by the automated segmentation of the Spectralis optical coherence tomography without ocular magnification adjustment.

Thickness Parameter	All Participants (N=164)	Control Group (AL<26mm; N=105)	High Myopic Group (AL≥26mm; N=59)	P-value*
**Total retina**				
Center subfield	270.5 ± 24.2	268.1 ± 25.0	277.2 ± 20.7	**0.044**
Temporal subfield	327.2 ± 18.3	326.1 ± 19.3	330.2 ± 14.9	0.222
Superior subfield	339.7 ± 18.6	338.5 ± 19.4	342.9 ± 15.8	0.194
Nasal subfield	341.8 ± 20.7	340.6 ± 21.2	345.0 ± 19.4	0.257
Inferior subfield	335.7 ± 19.9	334.5 ± 21.5	339.1 ± 14.5	0.244
**Retinal nerve fiber layer**				
Center subfield	11.7 ± 2.6	11.5 ± 2.7	12.5 ± 2.0	**0.022**
Temporal subfield	17.6 ± 1.6	17.6 ± 1.6	17.7 ± 1.7	0.713
Superior subfield	25.3 ± 4.5	24.4 ± 4.4	27.7 ± 3.9	**<0.001**
Nasal subfield	21.8 ± 3.5	21.2 ± 3.4	23.4 ± 3.5	**0.001**
Inferior subfield	25.3 ± 4.4	24.3 ± 4.1	27.9 ± 4.4	**<0.001**
**Ganglion cell layer**				
Center subfield	14.9 ± 5.2	14.2 ± 5.3	17.0 ± 4.1	**0.004**
Temporal subfield	45.8 ± 7.4	44.4 ± 7.4	49.6 ± 5.7	**<0.001**
Superior subfield	51.3 ± 6.4	50.5 ± 6.6	53.6 ± 5.2	**0.010**
Nasal subfield	49.9 ± 7.7	48.8 ± 8.1	52.9 ± 5.9	**0.004**
Inferior subfield	50.0 ± 7.7	49.0 ± 8.4	52.7 ± 4.2	**0.010**
**Inner plexiform layer**				
Center subfield	20.3 ± 3.9	19.9 ± 4.0	21.5 ± 3.3	**0.021**
Temporal subfield	40.4 ± 4.4	39.8 ± 4.4	42.1 ± 3.8	**0.004**
Superior subfield	40.2 ± 3.9	39.7 ± 4.0	41.4 ± 3.4	**0.022**
Nasal subfield	41.6 ± 4.6	40.8 ± 4.6	43.6 ± 4.0	**0.001**
Inferior subfield	39.9 ± 4.5	43.6 ± 4.0	41.1 ± 3.2	0.053
**Inner nuclear layer**				
Center subfield	19.8 ± 5.8	19.5 ± 6.3	20.7 ± 4.3	0.294
Temporal subfield	37.8 ± 3.7	38.0 ± 3.9	37.5 ± 2.9	0.448
Superior subfield	41.4 ± 4.0	41.8 ± 4.3	40.4 ± 2.9	0.066
Nasal subfield	41.6 ± 4.6	41.8 ± 4.8	41.1 ± 4.0	0.402
Inferior subfield	41.4 ± 4.7	41.4 ± 5.0	41.4 ± 4.2	0.968
**Outer plexiform layer**				
Center subfield	27.2 ± 7.5	26.4 ± 7.3	29.5 ± 7.9	**0.029**
Temporal subfield	34.2 ± 8.9	33.5 ± 9.0	35.8 ± 8.8	0.168
Superior subfield	38.2 ± 11.1	38.0 ± 11.1	38.6 ± 11.2	0.782
Nasal subfield	37.5 ± 11.2	37.5 ± 10.8	37.3 ± 12.4	0.922
Inferior subfield	38.2 ± 10.6	37.6 ± 10.4	39.6 ± 11.1	0.313
**Outer nuclear layer**				
Center subfield	88.7 ± 13.8	88.6 ± 14.5	89.0 ± 11.8	0.860
Temporal subfield	68.7 ± 11.3	69.5 ± 11.6	66.5 ± 10.0	0.157
Superior subfield	61.9 ± 13.6	62.2 ± 13.8	61.3 ± 12.9	0.710
Nasal subfield	66.6 ± 15.6	67.0 ± 15.2	65.4 ± 16.9	0.590
Inferior subfield	59.8 ± 13.7	61.0 ± 13.5	56.4 ± 13.8	0.072
**Photoreceptors**				
Center subfield	72.1 ± 4.2	72.5 ± 4.5	71.1 ± 2.9	0.073
Temporal subfield	68.0 ± 2.9	68.2 ± 3.2	67.3 ± 1.9	0.097
Superior subfield	66.3 ± 2.3	66.6 ± 2.5	65.6 ± 1.6	**0.025**
Nasal subfield	67.1 ± 2.9	67.3 ± 3.1	66.3 ± 2.1	0.058
Inferior subfield	66.3 ± 2.6	66.6 ± 2.8	65.6 ± 1.5	**0.040**
**Retinal pigment epithelium**				
Center subfield	16.5 ± 2.3	16.4 ± 2.4	16.7 ± 2.1	0.558
Temporal subfield	14.5 ± 1.6	14.6 ± 1.7	14.1 ± 1.1	0.098
Superior subfield	15.5 ± 2.0	15.7 ± 2.1	14.8 ± 1.2	**0.024**
Nasal subfield	15.3 ± 1.9	15.5 ± 1.9	14.7 ± 1.7	**0.033**
Inferior subfield	14.8 ± 2.0	15.0 ± 2.1	14.2 ± 1.3	**0.020**

AL, axial length.
Values are presented as mean thickness ± standard deviation; SD (μm).

P values were yielded by independent student t-test between the control group (AL<26mm) and high myopia group (AL≥26mm).

*Bold values indicate statistically significant differences (P<0.05).

Table S2. Effect of sex on individual retinal layer thicknesses and axial length prolongation before ocular magnification adjustment.

**Control Group (AL<26mm)**	**Male (n=55)**	**Female (n=50)**	**Mean difference**	**P-value***
Total retina	278.2 ± 22.1	253.1 ± 21.3	25.1 ± 4.3	**<0.001**
Retinal nerve fiber layer	12.6 ± 2.3	9.7 ± 2.2	3.0 ± 0.4	**<0.001**
Ganglion cell layer	15.3 ± 4.8	12.6 ± 5.6	2.7 ± 1.0	**0.008**
Inner plexiform layer	21.3 ± 3.8	17.8 ± 3.4	3.5 ± 0.7	**<0.001**
Inner nuclear layer	21.2 ± 5.9	17.1 ± 6.1	4.1 ± 1.2	**0.001**
Outer plexiform layer	27.3 ± 7.7	25.1 ± 6.4	2.1 ± 1.4	0.139
Outer nuclear layer	91.0 ± 16.4	85.0 ± 10.1	6.0 ± 2.8	**0.034**
Photoreceptors	72.8 ± 4.4	72.1 ± 4.7	0.7 ± 0.6	0.447
Retinal pigment epithelium	16.7 ± 2.0	16.0 ± 2.8	0.7 ± 0.5	0.125
**High Myopic Group (AL≥26mm)**	**Male (n=33)**	**Female (n=26)**	**Mean difference**	**P-value***
Total retina	279.2 ± 21.1	273.5 ± 14.5	5.7 ± 8.3	0.577
Retinal nerve fiber layer	12.5 ± 1.8	13.3 ± 2.5	-0.9 ± 0.8	0.261
Ganglion cell layer	17.1 ± 4.3	16.5 ± 3.5	0.6 ± 1.6	0.734
Inner plexiform layer	21.6 ± 3.4	21.3 ± 3.0	0.4 ± 1.3	0.784
Inner nuclear layer	20.5 ± 4.2	21.5 ± 4.6	-1.0 ± 1.7	0.555
Outer plexiform layer	29.7 ± 8.2	28.5 ± 6.9	1.2 ± 3.2	0.698
Outer nuclear layer	89.4 ± 11.0	87.5 ± 15.3	1.9 ± 4.7	0.688
Photoreceptors	71.1 ± 2.7	71.1 ± 3.8	-0.1 ± 1.2	0.959
Retinal pigment epithelium	17.2 ± 1.9	14.6 ± 1.7	2.6 ± 0.7	**0.001**

AL, axial length.
Values are presented as mean thickness ± standard deviation (μm).
P values were yielded by student t-test between male and female in each group divided by AL.
*Bold values indicate statistically significant differences (P<0.05).

Table S3. Effect of age on individual retinal layer thicknesses and axial length prolongation before ocular magnification adjustment by correlation analysis.

**Thickness parameter**	**Control Eyes (AL<26mm)**		**High Myopic Eyes (AL≥26mm)**	
	r	P*	r	P*
**Total retina**				
Center subfield	-0.197	**0.042**	0.182	0.267
Temporal subfield	-0.322	**0.001**	0.058	0.724
Superior subfield	-0.344	**<0.001**	0.034	0.837
Nasal subfield	-0.277	**0.004**	0.109	0.509
Inferior subfield	-0.362	**<0.001**	-0.091	0.580
**Retinal nerve fiber layer**				
Center subfield	-0.119	0.221	0.158	0.336
Temporal subfield	-0.278	**0.004**	-0.026	0.876
Superior subfield	-0.222	**0.022**	0.270	0.097
Nasal subfield	-0.204	**0.035**	0.065	0.694
Inferior subfield	-0.308	**0.001**	-0.084	0.610
**Ganglion cell layer**				
Center subfield	-0.224	**0.020**	0.055	0.740
Temporal subfield	-0.564	**<0.001**	-0.125	0.447
Superior subfield	-0.527	**<0.001**	-0.072	0.664
Nasal subfield	-0.488	**<0.001**	0.004	0.981
Inferior subfield	-0.570	**<0.001**	-0.144	0.383
**Inner plexiform layer**				
Center subfield	-0.175	0.071	0.043	0.793
Temporal subfield	-0.483	**<0.001**	-0.085	0.608
Superior subfield	-0.503	**<0.001**	-0.268	0.100
Nasal subfield	-0.444	**<0.001**	-0.226	0.167
Inferior subfield	-0.498	**<0.001**	-0.160	0.330
**Inner nuclear layer**				
Center subfield	0.026	0.792	0.313	0.052
Temporal subfield	-0.072	0.459	0.007	0.968
Superior subfield	-0.039	0.691	0.002	0.991
Nasal subfield	0.067	0.491	0.292	0.071
Inferior subfield	0.012	0.903	0.151	0.360
**Outer plexiform layer**				
Center subfield	-0.151	0.121	0.030	0.855
Temporal subfield	-0.220	**0.023**	0.287	0.076
Superior subfield	-0.069	0.482	-0.077	0.643
Nasal subfield	0.090	0.357	0.133	0.418
Inferior subfield	0.060	0.538	0.336	**0.034**
**Outer nuclear layer**				
Center subfield	-0.079	0.420	0.236	0.148
Temporal subfield	0.086	0.380	-0.103	0.531
Superior subfield	0.028	0.776	0.015	0.925
Nasal subfield	-0.053	0.586	0.033	0.841
Inferior subfield	-0.053	0.584	-0.189	0.249
**Photoreceptors**				
Center subfield	-0.100	0.305	0.157	0.341
Temporal subfield	-0.031	0.751	0.087	0.600
Superior subfield	-0.215	**0.026**	0.165	0.316
Nasal subfield	-0.173	0.075	0.091	0.581
Inferior subfield	-0.208	**0.031**	0.118	0.473
**Retinal pigment epithelium**				
Center subfield	-0.179	0.065	-0.518	**0.001**
Temporal subfield	0.128	0.187	-0.080	0.628
Superior subfield	0.081	0.407	-0.020	0.903
Nasal subfield	0.040	0.686	-0.168	0.307
Inferior subfield	0.031	0.749	-0.015	0.926

AL, axial length.

P values were yielded by partial correlation analysis (Pearson correlation) after adjusting for sex to evaluate the effect of age on macular thickness in each other group (r=partial correlation coefficients).

*Bold values indicate statistically significant differences (P<0.05).

Table S4. Correlation of the axial length with the thicknesses obtained by the OCT without the adjustment for ocular magnification.

**Center subfield**	r	95% CI	P
**Total retina**	0.330	0.187 to 0.459	**<0.001**
**Retinal nerve fiber layer**	0.245	0.096 to 0.383	**0.002**
**Ganglion cell layer**	0.347	0.205 to 0.474	**<0.001**
**Inner plexiform layer**	0.344	0.202 to 0.471	**<0.001**
**Inner nuclear layer**	0.197	0.047 to 0.340	**0.011**
**Outer plexiform layer**	0.302	0.157 to 0.435	**<0.001**
**Outer nuclear layer**	0.037	-0.116 to 0.188	0.635
**Photoreceptors**	-0.095	-0.243 to 0.059	0.226
**Retinal pigment epithelium**	0.096	-0.058 to 0.244	0.221

CI, confidential interval.
P values were yielded by Pearson correlation analysis to evaluate the effect of axial length on macular thickness (r= correlation coefficients).
*Bold values indicate statistically significant differences (P<0.05).
